# Shifts in human skin and nares microbiota of healthy children and adults

**DOI:** 10.1186/gm378

**Published:** 2012-10-10

**Authors:** Julia Oh, Sean Conlan, Eric C Polley, Julia A Segre, Heidi H Kong

**Affiliations:** 1 Genetics and Molecular Biology Branch, National Human Genome Research Institute, NIH, 49 Convent Dr., Bethesda, MD 20814, USA; 2 Biometric Research Branch, Division of Cancer Treatment and Diagnosis, National Cancer Institute, NIH, 6130 Executive Blvd, Rockville, MD 20852, USA; 3 Dermatology Branch, Center for Cancer Research, National Cancer Institute, NIH, 10 Center Dr., Bethesda, MD 20814, USA

## Abstract

**Background:**

Characterization of the topographical and temporal diversity of the microbial collective (microbiome) hosted by healthy human skin established a reference for studying disease-causing microbiomes. Physiologic changes occur in the skin as humans mature from infancy to adulthood. Thus, characterizations of adult microbiomes might have limitations when considering pediatric disorders such as atopic dermatitis (AD) or issues such as sites of microbial carriage. The objective of this study was to determine if microbial communities at several body sites in children differed significantly from adults.

**Methods:**

Using 16S-rRNA gene sequencing technology, we characterized and compared the bacterial communities of four body sites in relation to Tanner stage of human development. Body sites sampled included skin sites characteristically involved in AD (antecubital/popliteal fossae), a control skin site (volar forearm), and the nares. Twenty-eight healthy individuals aged from 2 to 40 years were evaluated at the outpatient dermatology clinic in the National Institutes of Health's Clinical Center. Exclusion criteria included the use of systemic antibiotics within 6 months, current/prior chronic skin disorders, asthma, allergic rhinitis, or other chronic medical conditions.

**Results:**

Bacterial communities in the nares of children (Tanner developmental stage 1) differed strikingly from adults (Tanner developmental stage 5). Firmicutes (Streptococcaceae), Bacteroidetes, and Proteobacteria (β, γ) were overrepresented in Tanner 1 compared to Tanner 5 individuals, where Corynebacteriaceae and Propionibacteriaceae predominated. While bacterial communities were significantly different between the two groups in all sites, the most marked microbial shifts were observed in the nares, a site that can harbor pathogenic species, including *Staphylococcus aureus *and *Streptococcus pneumonia*.

**Conclusions:**

Significant shifts in the microbiota associated with progressive sexual maturation as measured by Tanner staging suggest that puberty-dependent shifts in the skin and nares microbiomes may have significant implications regarding prevention and treatment of pediatric disorders involving microbial pathogens and colonization.

## Background

Studies of microbial diseases typically focus on individual pathogens as causative agents. Recent insights into the microbial communities inhabiting the human body suggest that pathogenicity can be enhanced or impaired by shifts in bacterial communities and that disturbance in the microbial ecosystem (dysbiosis) can contribute to disease [1]. Genomic sequencing surveys of the normal healthy human skin microbiome demonstrated vast complexity and diversity [2,3] influenced by both environmental and host factors. The influence of sexual maturation on the human skin microbiome is not well studied, but may have a profound effect on disease predilection and outcome.

Maturation-dependent shifts in the skin microbiome may be relevant to the prevention, diagnosis, and treatment strategies for disorders such as atopic dermatitis (AD) that differ in incidence and severity as individuals physiologically mature. Prior studies have characterized primarily cultivatable microbes [4,5] on the skin of subjects of different ages, but cultivation techniques have limited capacity to characterize fastidious or uncultivable organisms, which may represent upwards of 99% of bacteria [6]. In contrast, genomic studies, including metagenomic or 16S-ribosomal RNA sequence analysis, greatly increase the range of detectable microbes. The 16S-rRNA gene is present in all bacteria/archaea and contains both variable regions, which enable taxonomic classification, and conserved regions, which serve as universal binding sites for PCR primers. Such methods have been used to investigate the healthy adult [2,3] and the neonatal skin microbiomes [7,8], but differences in the composition and complexity of the skin microbiome as a result of major developmental stages such as puberty have not been explored.

Progression from infancy to adulthood encompasses major biological changes with significant physiologic and endocrinologic skin alterations that, in turn, likely influence the host-microbiome relationship. Since chronological age does not necessarily correspond with a defined stage in sexual maturation [9,10], use of Tanner staging of sexual maturity can provide a phenotypic assessment of the physiologic age of an individual. This study describes dramatic differences in the nares and skin microbiomes that occur in younger children (Tanner stage 1) versus adults (Tanner stage 5), with a smaller number of children in intermediate Tanner groups (stages 2 to 4) suggestive of a microbial shift occuring around puberty. Researchers should consider these physiologic differentials when designing and conducting investigations and developing interventions that promote healthy microbiomes.

## Materials and methods

### Subjects

Subjects were 28 healthy individuals (no systemic antibiotics within 6 months, no current or prior chronic skin disorders, chronic medical conditions, asthma, or allergic rhinitis, ascertained via an International Study of Asthma and Allergies in Childhood questionnaire [11]) ranging in age from 2 to 40 years (Table S1 in Additional file 1). Data were analyzed retrospectively from our previous skin survey of healthy adults [2] with the addition of two samples. Children (aged 2 to 17 years) were recruited into a prospective longitudinal study approved by the institutional review board of NHGRI, NIH for the study of children with AD and healthy age-matched controls [12] with written informed consent obtained from the parents or guardians of all participating minors. All subjects were recruited from the local Washington, DC metropolitan region. The Tanner staging of puberty [13,14] was determined by a board-certified physician (HHK). The study conformed to the ethical principles of the Declaration of Helsinki.

### Culture collection

Nasal *Staphylococcus aureus *cultures were performed by the Department of Laboratory Medicine (DLM), Clinical Center, NIH. Cultures were obtained with swabs (BBLtm CultureSwab™, made by Copan for Becton, Dickinson and Company, Sparks, MD, USA) and plated on three different growth media: chocolate agar, trypticase soy agar with 5% sheep blood, and mannitol salt agar (Remel Products, Lenexa, KS, USA). After overnight incubation at 37°C, colonies were counted and assessed macroscopically and also microscopically following staining by Gram's stain. Identification was confirmed using Staphaurex agglutination reagent (Remel Products) or standard coagulase test. Culture results were returned as an assignment to one of four categorical levels based on number of colonies per sample: 0 (zero), 1 (scant), 2 (light), 3 (moderate), or 4 (heavy), corresponding to 0, 1, 2 to 10, 11 to 40, or >40 colonies on a culture plate.

### Sample collection and processing

All samples were collected (HHK) as described [2,12]. Briefly, skin preparation instructions included avoidance of bathing for 24 hours, emollients, antimicrobial soaps, or shampoos for 7 days prior to all sampling. Sampling sites included the nares (N), antecubital fossa (Af), volar forearm (Vf), and popliteal fossa (Pf). From a 4 cm^2 ^area, bacterial swabs (via Epicentre swabs) and scrapes (via sterile disposable surgical blade) were obtained and incubated in enzymatic lysis buffer and lysozyme for 30 minutes at 37°C. For the nares, only swab samples were taken. Scrapes and swabs were obtained from the other sites as available. Correlation analysis of log proportions of genera identified in scrape versus swab sampling confirmed the similarity of techniques (R = 0.87, *P <*2.2 × 10^-16^, data not shown).

#### Preparation of samples for Sanger sequencing

For the Tanner stages 1 to 4 samples, genomic DNA was extracted with slight modifications to the original protocol used for the Tanner 5 subject samples [2,12], including a change in the enzymatic lysis buffer (Epicentre MPY80200) and lysozyme (Epicentre R1802M), increase in the frequency and the duration of bead-beating (30 Hz, 2 minutes), higher heating temperature of the sample (65**°**C), purification with Invitrogen columns, and reduced dilution of the eluate (to 35 μl). Full-length 16S-rRNA genes from genomic extractions were PCR amplified using the primer set 8F and 1391R, purified, cloned, and sequenced at the National Institutes of Health Intramural Sequencing Center (NISC) as described; 300 to 400 unique sequences were obtained from 16S amplicons of each clinical sample. To ensure that bias was not introduced by protocol modifications between the two studies, we validated our results on a subset of samples prepared identically and sequenced via both Sanger and 454 sequencing. The results strongly corroborated the results presented here (data not shown).

### Sequence analysis pipeline

Mothur v.1.21.0 [15] was used for sequence processing, definition of operational taxonomic units (OTUs), and downstream analyses. Sequence assembly, filtering, and alignment were performed as described [2,12]. Briefly, sequences matching the human genome were removed (E-value <0.1) then aligned using the Greengenes [16] NAST [17] aligner. Sequences were then chimera checked using the mothur implementation of UCHIME [18]. Sequences were classified using a Ribosomal Database Project naïve Bayesian classifier [19].

All uncorrected pairwise distances were calculated and OTUs defined at 97% similarity using average neighbor joining. No lane masking was applied. To estimate sampling saturation, rarefaction curves were generated for each site. Alpha diversity (community evenness and richness: Shannon diversity index, 'diversity'), and beta diversity (shared community structure/membership: theta, θ 'similarity' index) were calculated at a 97% similarity cutoff in mothur. To corroborate our OTU-based results, we also calculated phylogenetic trees using the clearcut implementation in mothur [20] (data not shown).

### Statistics

All data are represented as mean ± standard error of the mean unless otherwise indicated. Unless otherwise indicated, *P*-values were adjusted for multiple comparisons >6 using the p.adjust function in R using method='fdr' [21]. Statistical significance was ascribed to an alpha level of the adjusted *P*-values ≤0.05; adjusted values >0.05 and ≤0.1 were deemed to approach significance. Each site was treated as a separate dataset based on spatial physiological differences between different body niches [2]. All per-sample calculations are available upon request as a separate dataset. In general, analyses based on age rather than Tanner staging as a predictor variable were concordant for major taxa (data not shown).

For subjects with multiple measurements (for example, symmetric sites or multiple sampling timepoints), samples were processed independently and then pooled. Processed sequences were then subsampled at the smallest group size for that site (that is, number of Af sequences subsampled = 355, N = 328, Pf = 317, Vf = 321 and diversity statistics and proportions were calculated from the subsampled data. For alpha and beta diversity statistics, pools were subsampled 100× and resultant values averaged. Greater than 5 subsamplings yielded a 99.8% correlation with results from one subsampling, and 5 to 100 subsamplings yielded up to 99.99% similarity in value. Thus, for taxonomy-based statistics, we subsampled once. Similar results were obtained by averaging symmetric sites and using a mixed effects model with a random intercept for multiple timepoints (data not shown). Analysis of variance (ANOVA) and analysis of molecular variance (AMOVA) [22] were used with θ to determine statistically significant differences between microbial populations at different Tanner stages.

For linear regression analysis of alpha diversity of the subsampled data, Tanner stage was used as a five-level factor variable. For all subsequent analyses, we performed two sets of comparisons. First, we performed comparisons between Tanner stages 1 and 5, which comprised the largest sample sizes. Second, we generated statistics for grouped Tanner stages 1 to 3 ('Tanner1-3') and Tanner stages 4 to 5 ('Tanner4-5') due to strong segregation of Tanner stages 2 and 3 with Tanner stage 1, and Tanner stage 4 with 5 during principal coordinates analyses. For beta diversity metrics, means and ANOVA were performed on the subsampled data and Tukey's honest significance test was used for all *post hoc *comparisons. To analyze the over/underrepresentation of taxonomies between different Tanner stage groups, we used a linear model using Tanner staging as a two-level predictor variable and the subsampled proportions as the dependent variable. We performed this regression on the proportions obtained using i) 12 major phyla/family categories, ii) OTUs classified at the genus level, and iii) species (*Staphylococcus *and *Streptococcus *only, for which we used custom scripts and databases to speciate).

To determine if the relative abundance of *S. aureus *in the nares was correlated with the relative abundance of *S. aureus *at other skin sites, we calculated the Spearman correlation coefficients between the relative abundances of the nares and the skin sites. To determine if presence or absence of *S. aureus *in the nares was consistent with the presence or absence of *S. aureus *≥1 skin site, we performed a Pearson's Chi-squared test for count data.

### Accession numbers

Sequence data have been deposited in GenBank under the accession numbers [GQ000001] to [GQ116391] and can be accessed through BioProject ID 46333.

## Results

To determine if microbial communities in children differed significantly from adults, we analyzed a total of 114,410 full-length 16S-rRNA sequences for 4 skin/nares sites in 28 individuals. Rarefaction curves indicated that our sampling provided sufficient coverage to analyze dominant members of the bacterial communities (Figure S1 in Additional file 2). Because of our interest in the role of the skin microbiome in diseases such as AD, we analyzed data obtained from symmetric skin sites relevant to disease predilection: the antecubital fossae (Af) and popliteal fossae (Pf), the volar forearm (Vf, a control for these two sites), and the nares (N), a potential reservoir for pathogens such as *S. aureus *and *S. pneumoniae *[23].

### Comparisons between Tanner stages

Tanner staging is a standardized measurement of assessing pubertal development based on physiological characteristics, specifically primary and secondary external sex characteristics [13,14]. Due to natural variability in sexual maturation rates, Tanner stages are considered more accurate assessments of maturation than chronological ages.

To assess whether there is a shift in the microbial composition of children (Tanner stage 1) versus adults (Tanner stage 5), we calculated the variation between individuals within and between the two groups. θ index is a measure of overall microbial community similarity with values between 0 and 1: a value of 1 implies identical community structure, and a value of 0 implies dissimilar community structures.

**Figure 1:**
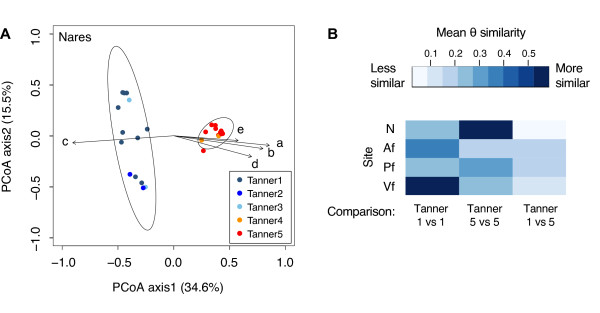
**Microbial community-level statistics**. **(a) **Nares samples clustered using principal coordinates analysis (PCoA) of the θ 'similarity' coefficient, which calculates similarity between two samples based on i) number of species in common between two samples and ii) their relative abundances. Colors represent different Tanner stages. Samples that have similar principal coordinates appear closer together, that is, are more similar. Ellipses represent the 95% confidence intervals based on standard deviation. Biplot arrows indicate the five most significant unique consensus taxonomies contributing to variation along axis 1. (Spearman correlations with axes and associated *P-*values are shown in Table S3 in Additional file 1). a, *Propionibacterium*; b, *Corynebacterium*; c, *Streptococcus*; d, *Turicella*; e, *Anaerococcus*. Additional skin sites are shown in Figure S2 in Additional file 2. **(b) **Heatmap of mean θ similarity coefficients of pairwise comparisons of communities within or between individuals of Tanner stage 1 and Tanner stage 5 (within-group, Tanner 1 versus Tanner 1 or Tanner 5 versus Tanner 5; between-group, Tanner 1 versus Tanner 5). Darker color indicates higher similarity: θ = 0 indicates that two samples have no species in common and θ = 1 indicates that two samples are identical. Site abbreviations: Af, antecubital fossa; N, nares; Pf, popliteal fossa; Vf, volar forearm.

We performed principal coordinates analysis (PCoA) based on the θ 'similarity' index, where a shorter distance between points indicates increasing similarity (Figure 1a; Figure S2 in Additional file 2). Biplot arrows show the most significant taxa contributing to axis variation; these taxa overlapped between the three skin sites and nares (Table S2 in Additional file 1). The nares microbiome at Tanner stages 1, 2, and 3 strongly segregated from the nares microbiome of Tanner stages 4 and 5, forming two distinct clusters with significantly different centroids (AMOVA for the two centroids, *P *< 0.001). This separation was less marked but statistically significant in the three skin sites (AMOVA *P *< 0.001; Figure S2 in Additional file 2).

Since sample sizes for intermediate Tanner stages (2, 3, and 4) were small, we assessed community differences between the stages with the largest numbers of subjects (Tanner stage 1 versus 5). To investigate trends that might occur during the physiological transition from childhood to adulthood, we also examined all of the subjects separated into two groups: Tanner stages 1 to 3 ('Tanner1-3') and Tanner stages 4 to 5 ('Tanner4-5') based on the strong segregation of Tanner stages 2 and 3 with Tanner stage 1, and Tanner stages 4 with 5 observed in the PCoA analysis. We reported both analyses because the initial analysis of the two largest cohorts demonstrated significant differences between Tanner stages 1 (young children) and 5 (adults), and the latter analysis included a smaller number of individuals in intermediate Tanner stages, suggesting that a shift occurs in the skin and nares microbiome during the physiological transition from childhood to adulthood.

**Figure 2:**
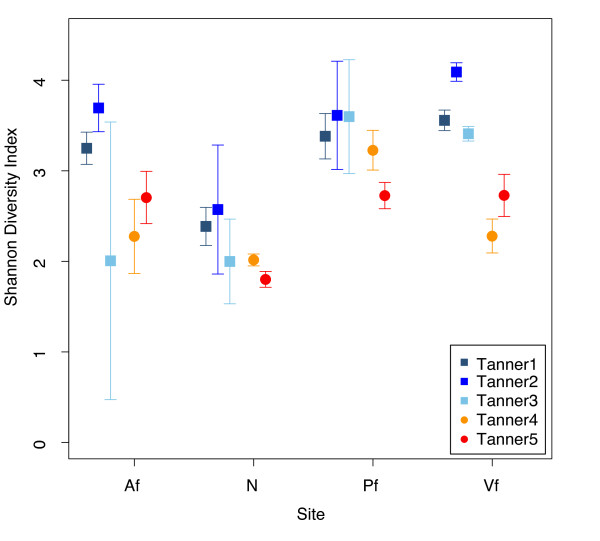
**Mean Shannon diversity ± SEM for each Tanner stage group (1-5) at all sites**. Squares (blue hues) show data from Tanner stages 1 through 3; circles (red hues) indicate data from Tanner stages 4 and 5. Site abbreviations: Af, antecubital fossa; N, nares; Pf, popliteal fossa; Vf, volar forearm.

Within the nares, the interpersonal variation between children was θ = 0.269 ± 0.035 and between adults was θ = 0.585 ± 0.023 (Figure 1b). In contrast, the mean similarity between children and adults was only θ = 0.025 ± 0.003 (Figure 1b), demonstrating significantly greater similarity within the groups than between the two groups. More generally, the mean θ similarity of the Tanner 1 microbial communities differed significantly from that of the Tanner 5 microbial communities at the different skin sites (Figure 1b), exceeding baseline levels of interpersonal variation (Table S3 in Additional file 1; ANOVA 2.2 × 10^-16 ^<*P <*8.1 × 10^-3^; Af Tanner 5 versus interpersonal variation not significant). Interestingly, interpersonal variation between Tanner 5 individuals was lower than Tanner 1 individuals in the nares (*P *< 2.2 × 10^-16^). These trends were correspondingly significant in 'Tanner1-3' and 'Tanner4-5' groups. Furthermore, the observation that microbial 'diversity' of the nares microbiome decreased with increasing sexual maturity (*P *= 7.8 × 10^-03^; Figure 2; Table S4 in Additional file 1) suggests a stabilization and convergence of the nares microbiome in more mature individuals. Finally, we verified that these microbial community differences were not caused by gender-related differences; θ 'similarity' between males and females at all sites did not differ significantly regardless of Tanner stage (*P *> 0.05; Table S3 in Additional file 1).

### Bacterial taxonomies significantly associated with Tanner stage

**Figure 3:**
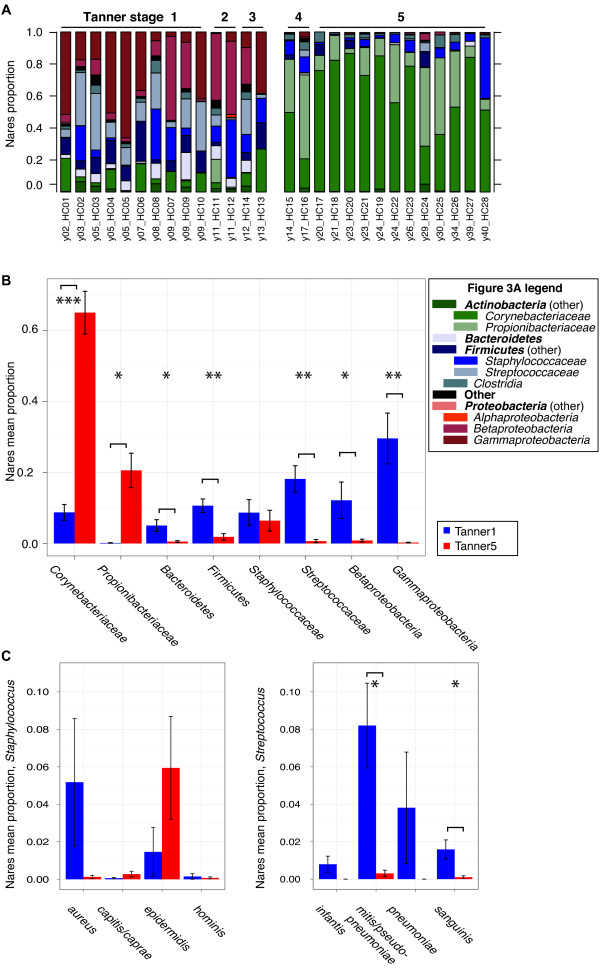
**Bacterial taxonomic classifications and comparisons of relative abundance between Tanner stage groups**. **(a) **Relative abundances of 12 major phyla-family taxonomies in nares. Subjects are ordered by age ('y**') and Tanner staging is indicated. **(b) **Barplots of mean proportions of select major phyla-family classifications in nares. Statistical significance for false-discovery rate-adjusted *P*-values was assessed at α ≤ 0.05; *P ≤ 0.05, **P ≤ 0.001, ***P ≤ 0.0001. Tanner 1 means are shown in blue; Tanner 5 in red. The full version for the nares is provided in Figure S4 in Additional file 2. **(c) **Barplots of mean proportions of major species designations for genera *Staphylococcus *(left) and *Streptococcus *(right) in nares; descriptions are as in (b).

To identify bacteria distinguishing Tanner 1 from Tanner 5 microbiomes, we classified the taxonomy of the sequence data; proportions of major taxa are shown in Figure 3a and Figure S3 in Additional file 2. We compared relative abundances of each bacteria at several taxonomic levels to determine differences between Tanner stages 1 and 5 at: i) a high phylum-family level; ii) a more specific genus level; and then where possible, at iii) the species level to determine the prevalence of opportunistic pathogens such as *S. aureus *and *S. pneumoniae*. Results comparing 'Tanner1-3' and 'Tanner4-5' groups, which were generally highly concordant with Tanner stages 1 versus 5 comparisons, are also reported in Tables S5 to S8 in Additional file 1.

#### High-level analysis (phylum-family)

The nares microbiomes in Tanner stage 1 individuals were primarily composed of Streptococcaceae and other Firmicutes, Bacteroidetes, and β- and γ-Proteobacteria, while the nares microbiomes in Tanner stage 5 were strikingly dominated by Corynebacteriaceae and Propionibacteriaceae (Figure 3b, Table 1). This pattern of differential dominant phyla identified in younger children and adults was generally consistent across the three skin sites studied (antecubital fossa, volar forearm, and popliteal fossa; Figures S4 in Additional file 2; Table S5 in Additional file 1).

**Table 1:** Abundant taxonomies differentially present in the nares

**Classification: phylum/class/family/genus^a^**	**Tanner 1 (mean ± SE)**	*	**Tanner 5 (mean ± SE)**	**Adjusted *P*-value**
**Actinobacteria **(other)	2.1 ± 0.7%	≈	0.8 ± 0.4%	0.154^b^
Corynebacteriaceae	8.8 ± 2.2%	<<	65.0 ± 6.0%	1.18E-06
Propionibacteriaceae	0.2 ± 0.1%	<<	20.6 ± 4.8%	0.0027
*Corynebacterium*	8.7 ± 2.2%	<<	49.6 ± 5.6%	0.0001
*Propionibacterium*	0.1 ± 0.1%	<<	20.6 ± 4.8%	0.0055
*Rothia*	1.4 ± 0.5%	>	0.1 ± 0.1%	0.0249
*Turicella*	0.0 ± 0.0%	<<	15.4 ± 4.2%	0.0119
**Bacteroidetes **(other)	5.1 ± 1.6%	>>	0.5 ± 0.3%	0.0158
*Capnocytophaga*	0.7 ± 0.2%	>	0.0 ± 0.0%	0.0007
*Paraprevotella*	0.7 ± 0.2%	>	0.0 ± 0.0%	0.0069
*Porphyromonas*	1.4 ± 0.5%	>	0.2 ± 0.2%	0.041
*Prevotella*	0.6 ± 0.2%	>	0.1 ± 0.0%	0.0201
**Firmicutes **(other)	10.6 ± 1.9%	>>	1.9 ± 0.9%	0.0009
Staphylococcaceae	8.7 ± 3.7%	>	6.4 ± 2.9%	0.6836^b^
Streptococcaceae	18.2 ± 3.7%	>>	0.7 ± 0.5%	0.0003
Clostridia	2.7 ± 0.8%	≈	2.8 ± 0.7%	0.9342^b^
*Dolosigranulum*	8.6 ± 2.2%	>>	1.7 ± 0.9%	0.0144
*Gemella*	1.7 ± 0.6%	>	0.0 ± 0.0%	0.0143
*Granulicatella*	1.2 ± 0.4%	>	0.0 ± 0.0%	0.0229
*Streptococcus*	18.1 ± 3.7%	>>	0.7 ± 0.5%	0.0006
**Fusobacteria**				
*Fusobacterium*	0.6 ± 0.3%	>	0.0 ± 0.0%	0.0403
**Proteobacteria **(other)	0.1 ± 0.1%	≈	0.0 ± 0.0%	0.3356^b^
Alphaproteobacteria	0.3 ± 0.1%	>	0.1 ± 0.0%	0.0317
Betaproteobacteria	12.2 ± 5.1%	>>	0.8 ± 0.4%	0.0365
Gammaproteobacteria	29.6 ± 7.2%	>>	0.3 ± 0.1%	0.0009
*Neisseria*	2.3 ± 0.9%	>	0.1 ± 0.1%	0.0201
*Haemophilus*	3.7 ± 1.5%	>	0.0 ± 0.0%	0.0256
*Moraxella*	24.1 ± 7.0%	>>	0.0 ± 0.0%	0.0055

^a^Represents proportion in the microbiome as a whole, not a subset of the taxonomy. Higher taxonomic levels are listed first. ^b^Not significant; see Additional file 2 for additional details. *Greater or less than; double signs indicate a difference of notable magnitude.

#### Genus-level analysis

In Tanner stage 1, Firmicutes *(Streptococcus*, *Dolosigranulum*, *Gemella*, and *Granulicatella) *and Proteobacteria (*Moraxella*, *Haemophilus*, and *Neisseria*) were the significantly overrepresented major genera in the nares (Table 1; Table S6 in Additional file 1). Actinobacteria (particularly *Corynebacterium*, *Propionibacterium*, and *Turicella*) were overrepresented in the nares of Tanner stage 5 individuals. Similar trends for many of these genera were observed for the three skin sites when comparing Tanner stages 1 versus 5, and 'Tanner1-3' versus 'Tanner4-5'.

#### Species level analysis

We further classified *Streptococcus *and *Staphylococcus *sequences by alignment to a curated reference database (Figure 3c; Figure S5 in Additional file 2; Tables S7 and S8 in Additional file 1). The commensal *Streptococcus mitis/ Streptococcus pseudopneumoniae *dominated streptococcal sequences overrepresented in children in all sampled sites, along with an overrepresentation of *Streptococcus sanguinis *and *Streptococcus salivarius*. Although overrepresentation of *S. aureus *and other staphylococci did not reach statistical significance in the Tanner 1 and Tanner 5 comparison due to high variability in relative abundance in the nares, *S. aureus *was significantly overrepresented in the 'Tanner1-3' nares compared with those of the older 'Tanner4-5' group.

### Comparison with culture-based assessments and *S. aureus *as a reservoir of colonization

**Figure 4:**
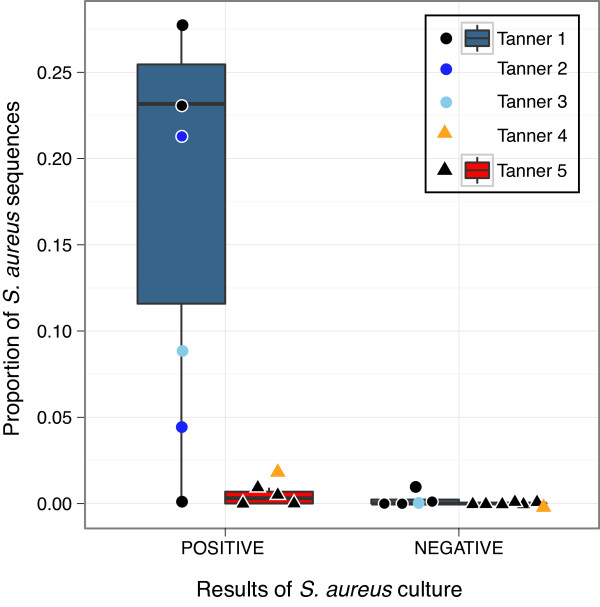
**Comparison between *S. aureus *culture and 16S-rRNA sequencing**. Cultures were scored based on the following criteria: zero colonies of *S. aureus *(negative for *S. aureus*) and ≥1 colony (positive for *S. aureus*). The boxplot represents the nares culture results for *S. aureus *compared to the sequence-based relative abundance values of *S. aureus*. Tanner 1 group in gray-blue; Tanner 5 in red. Points for additional Tanner stages are plotted but do not contribute to the boxplot dimensions.

Given the potential of *S. aureus *as a pathobiont with the nares as a major reservoir for infection and persistence, each subject's nares sample was concurrently tested for presence and sensitivities of *S. aureus *via traditional culture-based techinques. Presence of *S. aureus *(see Materials and methods) was significantly associated with relative abundance of *S. aureus *nares genomic sequences (*ρ *= 0.73, *P = *9.1 × 10^-05^) across individuals of all Tanner stages, validating that relative abundance of genomic sequences is related to culture-based bacterial bioburden. *S. aureus *bioburden in the nares of Tanner 1 children was not significantly higher than in Tanner 5 adults (Figure 4; Table S10 in Additional file 1; Wilcoxon rank-sum test, *P = *0.41). This was in contrast to the same comparison with sequence-based *S. aureus *relative abundances, with levels approaching significance (*P = *0.11) for Tanner 1 versus Tanner 5, and reaching significance in the 'Tanner1-3' versus 'Tanner4-5' comparison (*P *= 0.05). These results suggest a trend of higher *S. aureus *relative abundance in younger children, with discordance with culture-based bioburden arising from either the higher resolution afforded by 16S-rRNA sequencing, or from the fact that bioburden is not necessarily an analogous reflection of relative community composition. None of the subjects sampled tested positive for methicillin-resistant *S. aureus*, suggesting that while *S. aureus *may be more abundant and occur more frequently in younger populations, there is no indication that carriage of methicillin resistance is dependent on age in our cohort.

Finally, to determine if the presence of *S. aureus *in the nares could serve as a reservoir for colonization to other skin sites, we determined if the incidence of *S. aureus *in the nares was associated with incidence in at least one of three distant skin sites. Across Tanner stages, we found a significant association between the incidence in the nares and incidence at other skin sites (Χ^2 ^(1, *N *= 26) = 9.2, *P = *0.002). We also determined that relative abundances of *S. aureus *in the nares and the distant skin sites were significantly correlated (Af, Spearman correlation ρ = 0.53, *P = *0.006; Pf, ρ = 0.61, *P=*0.001; Vf, ρ = 0.58, *P=*0.002). However, this trend was generally non-specific to Tanner 1 or 5 (Χ^2 ^(1, Tanner 1 *N *= 10, Tanner 5 *N *= 10 for 'Tanner4-5') = 0.82, 1.1; *P = *0.37, 0.30, respectively), and so we conclude that these data are consistent with the concept that the nares can serve as a reservoir for *S. aureus*, regardless of age.

## Discussion

We identified a clear dichotomy in the skin and nares microbiomes between Tanner stage 1 children and Tanner stage 5 adults. Including a smaller number of subjects grouped as Tanner stages 2 to 4, we observed that the microbial community memberships and structures of individuals of Tanner stages 2 and 3 trended towards more significantly resembling those of Tanner stage 1 than later stages, even though Tanner stages 2 and 3 are considered early stages of puberty. Physiologically, Tanner 4 individuals more closely resemble Tanner 5 individuals in terms of sexual maturity [13,14], sebum production [24], and the presence of other skin surface lipids in the face [25]. Correspondingly, we found that the microbial communities clustered into two distinct groups in which those of Tanner stages 1, 2, and 3 differed significantly from those of Tanner stages 4 and 5. Thus, we compared not only the microbiomes of Tanner 1 and Tanner 5 individuals, but also the microbiomes of two physiological groupings, young children ('Tanner1-3') and adolescent/post-adolescent individuals ('Tanner4-5'). We observed that a greater diversity of bacteria, including *Streptococcus *and Gram-negatives *Moraxella*, *Haemophilus*, and *Neisseria*, dominated the microbiomes of Tanner stage 1 children. In contrast, Tanner stage 5 individuals had few to none of these taxa; their microbiomes were dominated instead by lipophilic bacteria, including *Propionibacterium*, *Corynebacterium*, and *Turicella*, that are associated with sebaceous skin regions [26,27]. The results comparing Tanner stages 1 to 3 versus stages 4 to 5 were highly concordant.

We attribute the prevalence of lipophilic taxa to increased epidermal lipids resulting from increased hormone-stimulated sebaceous gland activity during puberty. Higher sebum concentration markedly favors colonization by lipophilic bacteria at the expense of other flora - metabolism of skin lipids reduces skin pH, which in turn inhibits staphylococcal and streptococcal species [28,29]. Other skin changes that occur during puberty include increased density and thickness of body hair and increased apocrine gland activity, which may also contribute to the observed changes in microbial communities. Alternatively, subsets within the larger immune system, possibily immune components in skin, may be less mature in younger individuals and may permit the colonization and growth of a wider range of bacteria, consistent with the higher community-wide diversity we observed in younger children.

Samples from characteristially sebaceous sites, such as the head or upper torso, have the highest density of sebaceous glands and have been shown to have a different microbiome from less sebaceous sites [2]. The skin sites included in this study do not typically have the highest density of sebaceous glands. However, the antecubital/popliteal fossa and volar forearms contain hair follicles that develop sebaceous glands during puberty, but to a lesser degree than skin sites classically defined as sebaceous. It is possible, as suggested in our study, that even moderate changes in lipids on the skin may be sufficient to drive colonization of lipophilic bacteria in the skin.

We did not observe significant gender-related differences associated with numerous sexually dimorphic traits that occur during puberty. For example, production and metabolism of sex hormones cause differential production and composition of sebum between males and females [24,30]. Again, even modest increases in lipid production may be sufficient to alter microbiome colonization and composition. Alternatively, previous studies characterizing sebum production have focused on the forehead, a sebaceous site where the density of sebaceous glands can outnumber those at other body sites by over 10-fold [30]. Moist and dry skin sites, such as those surveyed in our study, may thereby exhibit less sexual dimorphism. Another possibility is that there may be differences in the absolute abundances of bacteria, as there have been reports of higher bacterial loads on male adult skin compared with that of females [31], but the relative compositions of the microbiota are similar between gender.

While all sites surveyed showed distinct maturation-related microbial differences, this contrast was particularly striking in the nares, which can serve as a reservoir for potential pathogens, including *S. aureus *and *S. pneumoniae *[23]. Studying *S. aureus *in the context of its microbial communities will facilitate understanding how microbial communities contribute to the initiation and perpetuation of skin disease. For example, AD is a primarily pediatric disease that often resolves by adolescence and adulthood. We observed an elevated incidence and abundance of *S. aureus *in the nares of the 'Tanner1-3' group, which may be a factor in the higher incidence, recurrence, and severity of AD observed in this age group. Our findings do not necessarily explain the resolution of AD, particularly in those patients whose symptoms improve before puberty. However, AD is a complex inflammatory disease, and human-associated microbial communities may be a contributory factor.

Moreover, our results demonstrate that *S. aureus *abundance and incidence in the nares was significantly associated with those found at distant skin sites. Of note, this study cannot assess the directionality of colonization. However, staphylococcal and streptococcal species in the nares are replaced by lipophilic and other bacteria during late/post-adolescence - a potential contributor to the age-related reduction in incidence and severity of skin disorders, such as AD. Lipophiles, once established in the skin and nares of mature individuals, may create an inhospitable environment for staphylococci, thus reducing both prevalence as well as bioburden of AD and *S. aureus*. These findings raise an interesting evolutionary question regarding host-microbiome interactions that may reduce the ability to harbor potential pathogens upon reaching reproductive age.

In all Tanner stages examined, the skin sites in these healthy subjects were significantly more similar to each other as compared to the nares. The relative similarity of skin sites is consistent with prior studies that showed that samples clustered based on the body sites of gut, skin, vagina, and oral cavity [3,32]. These findings are in contrast to patients with atopic dermatitis, who demonstrate a distinct difference in sites more characteristically affected with skin disease, such as the antecubital fossa compared to the volar forearm [12].

The significant shift in elevated relative abundances of *Corynebacterium*, *Propionibacterium*, and other lipophiles in the nares and increased prevalence in the flexural sites (antecubital, popliteal fossa) and even non-flexural sites (volar forearm) with sexual maturation was unexpected. Maturation of sebaceous glands and sebum secretion in early childhood may progress asynchronously across body sites, occurring earliest at typically sebaceous and proximal skin regions, including the face, back, and chest. Transition into a more sexually mature microbiome at distal and less characteristically sebaceous regions may be delayed. The delayed maturation at these sites may contribute to the persistence into adolescence of diseases that are linked to staphylococcal species, given the antagonism of lipophilic species and staphylococci [28,29].

While our results generally corroborate lower-resolution culture-based methods showing increased diversity in the skin flora of children and increased colonization by diptheroids at other skin sites in adults [4,5], genomics-based methods offer more comprehensive detection and quantification of both cultivatable and fastidious organisms. Results from the culture-based methods may be skewed towards fast-growing organisms and are most useful for incidence-based studies as they give less information on the relative abundance of a taxa in the microbial community as a whole. For example, we observed trace, if any, amounts of *Sarcina *in either children or adults as reported by culture-based studies [4,5], and *Neisseria*, which we observed to be significantly overrepresented in the 'Tanner1-3' group, occurred in 'high incidence' in adults as well as children. A likely explanation for this discrepancy is that these studies tested different body sites, such as the forehead, toewebs, hands, and ear. Previous work from our laboratory and others has demonstrated great taxonomic diversity between different body sites [2,3], despite having similar physiological characteristics like sebaceous, moist, or dry microenvironments. These culture-based studies also classified diptheroids as 'high-incident' in both children and adults, which we corroborate; however, we show that their representation in the microbial communities is significantly diminished in younger indiviuduals. Finally, our study contrasts with previous work examining the normal flora of skin in different age groups in that we focused on sites with clinical predilection to diseases such as AD, such as the antecubital and popliteal fossae, in which these dynamics between liphophiles and potential pathogenic genera such as *Staphylococcus *and *Streptococcus *are of particular clinical interest and may be exploited for preventative or therapeutic (pre/probiotic) approaches.

## Conclusions

Future studies will further define microbial interactions, disease-causing perturbations to microbial ecology, and host-commensal microbe relationships. Moreover, therapies based on probiotics or altering the balance of microbial communities have the potential to augment or bypass antibiotic use, decreasing the rise of antibiotic-resistant bacteria. Our results from this genomic microbial survey demonstrate a significant difference in the microbiomes of children versus adults at body sites relevant to infections and inflammatory skin diseases. These microbiome differences must be considered in the design and interpretation of studies concerning the etiology, prevention, and treatment of skin diseases with microbial etiology or influence.

## Abbreviations

AD: atopic dermatitis; Af: antecubital fossa; AMOVA: analysis of molecular variance; ANOVA: analysis of variance; N: nares/nostril; OTU: operational taxonomic unit; PCoA: principal coordinates analysis; Pf: popliteal fossa; Vf: volar forearm.

## Competing interests

The authors declare that they have no competing interests.

## Authors' contributions

Study concept and design: JO, JAS, HHK. Acquisition of data: JO, HHK. Drafting of the manuscript: JO, JAS, HHK. Critical revision of the manuscript for important intellectual content: SC, ECP, JAS, HHK. Statistical analysis: JO, ECP. Administrative, technical, or material support: SC, ECP, JAS, HHK. Study supervision: JAS, HHK. All authors read and approved the manuscript.

## Supplementary Material

Additional file 1 **Additional Tables S1 to S7**. Table S1: summary of subject characteristics. Table S2: top 20 most significant unique consensus taxonomies indicated in biplots in Figure 1 and Figure S2 in Additional file 2 with Spearman correlations with axes and associated *P-*values. Table S3: theta similarity indices and statistical testing for subsampled (n = 100) values. Table S4: Shannon diversity indices and statistical testing on subsampled (n = 100) data. Table S5: statistical testing for proportions of higher-order classifications, all sites. Table S6: genera significantly over/underrepresented between groups, all sites. Table S7: species-level classification summary for *Streptococcus *and statistical testing for over/underrepresentation. Table S8: species-level classification summary for *Staphylococcus *and statistical testing for over/underrepresentation. Table S9: culture data and proportion of *S. aureus *for the nares. Click here for file

Additional file 2 **Additional Figures S1 to S5**. Figure S1: rarefaction analysis for the skin microbiota sampling at each site (Af, Pf, Vf, and N) calculated as operational taxonomic units (OTUs) at a cutoff of 97% similarity. Each point represents mean ± standard error of the mean of all individuals at the specified site and Tanner stage. Figure S2: communities clustered using principal coordinates analysis (PCoA) of the theta 'similarity' coefficients at all sites. Biplot arrows indicate the five most significant unique consensus taxonomies contributing to variation along axis 1. Spearman correlations with axes and associated *P-*values are shown in Table S3 in Additional file 1. Sites indicated are: Af, antecubital fossa; Pf, popliteal fossa; Vf, volar forearm. The nares plot is shown in Figure 1a. Analysis of molecular variance (AMOVA) testing differences in centroids is indicated for both Tanner 1 versus Tanner 5 and 'Tanner1-3' versus 'Tanner4-5'. Figure S3: bacterial taxonomic classifications for additional sites. Tanner stage is indicated. Figure S4: mean relative abundances by Tanner group for 12 major phyla-family taxonomic classifications. *P ≤ 0.05, **P ≤ 0.001, ***P ≤ 0.0001. Tanner 1 in red; Tanner 5 in blue. Figure S5: mean relative abundances by Tanner group for major species designations for genera *Streptococcus *and *Staphylococcus*. *P ≤ 0.05, **P ≤ 0.001, ***P ≤ 0.0001. Tanner 1 in red; Tanner 5 in blue. Click here for file
