# Supplementary figures and images for: Shifts in human skin and nares microbiota of healthy children and adults
Source: Genome Med. 2012 Oct 10;4(10):77. doi: 10.1186/gm378 (PMC3580446; doi:10.1186/gm378)

Additional File 1, Figure S1

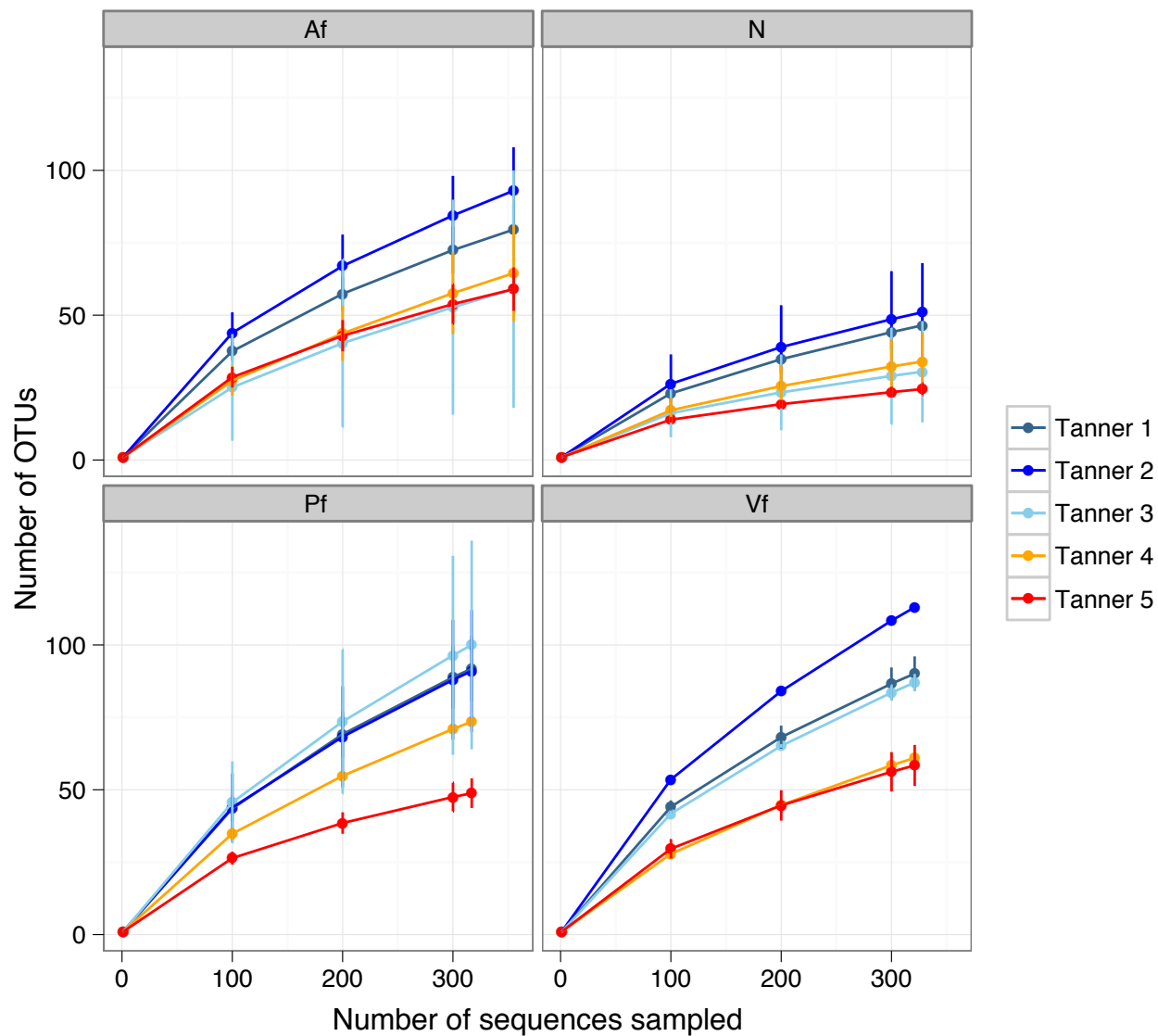

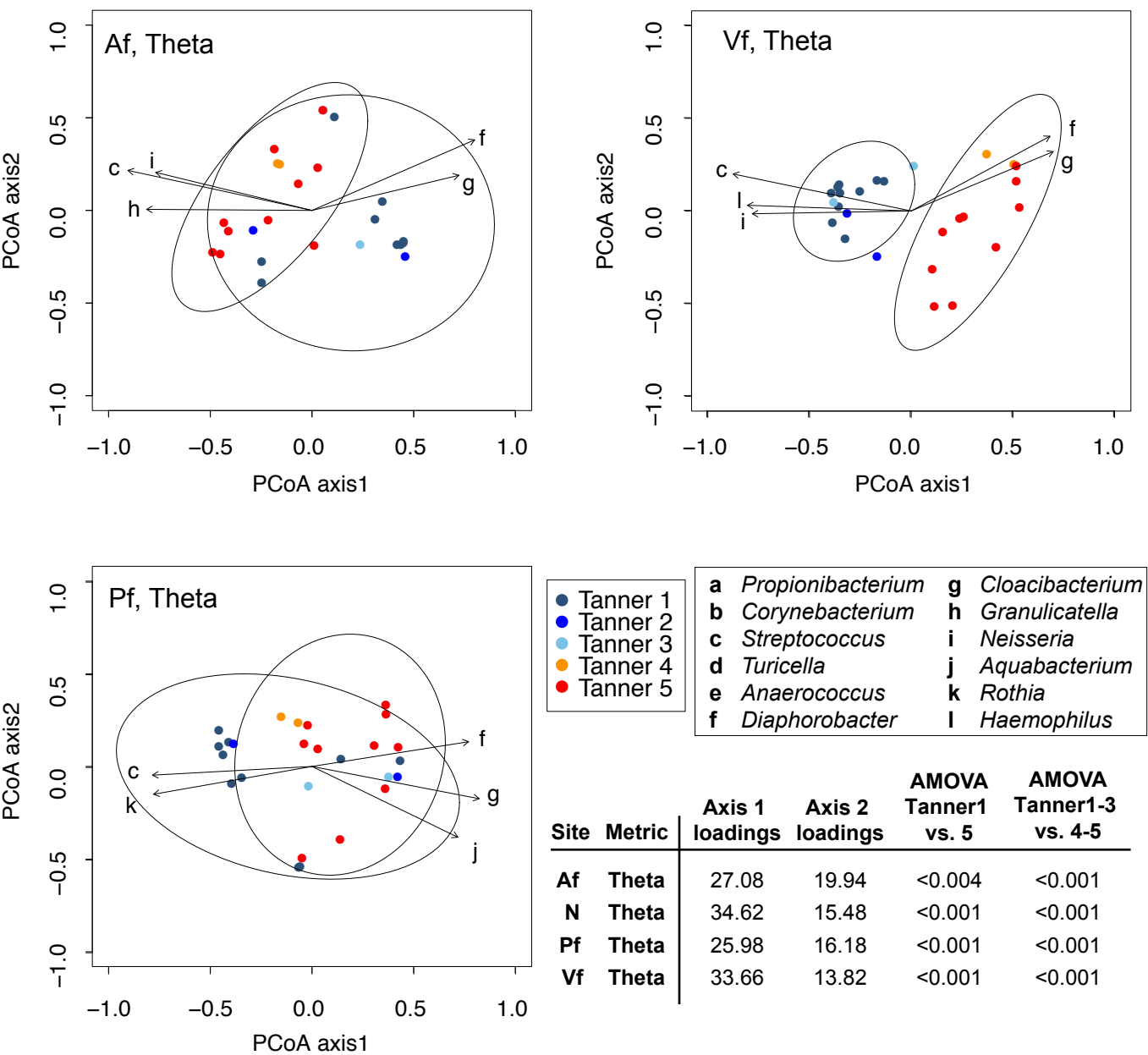

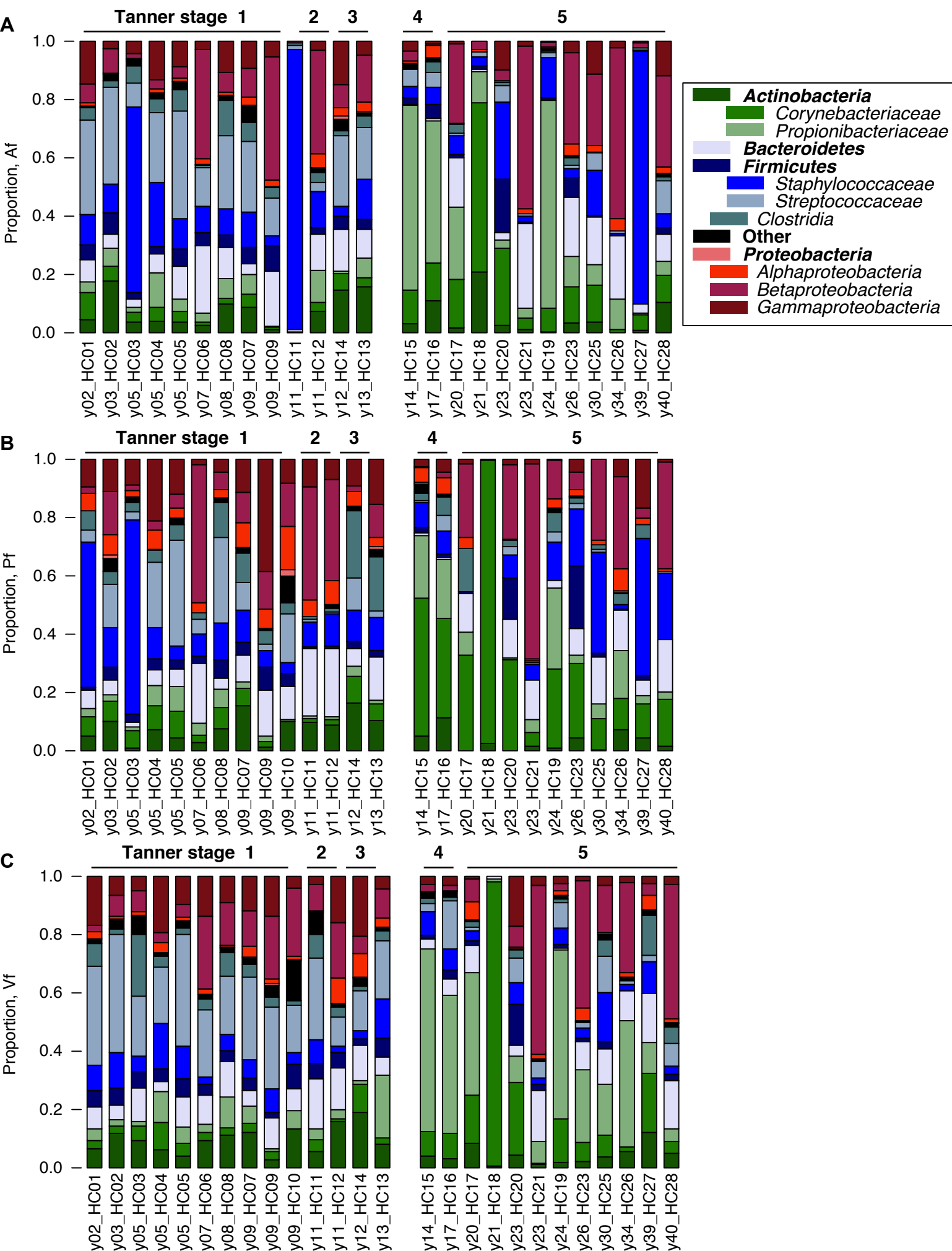

Additional File 1, Figure S4

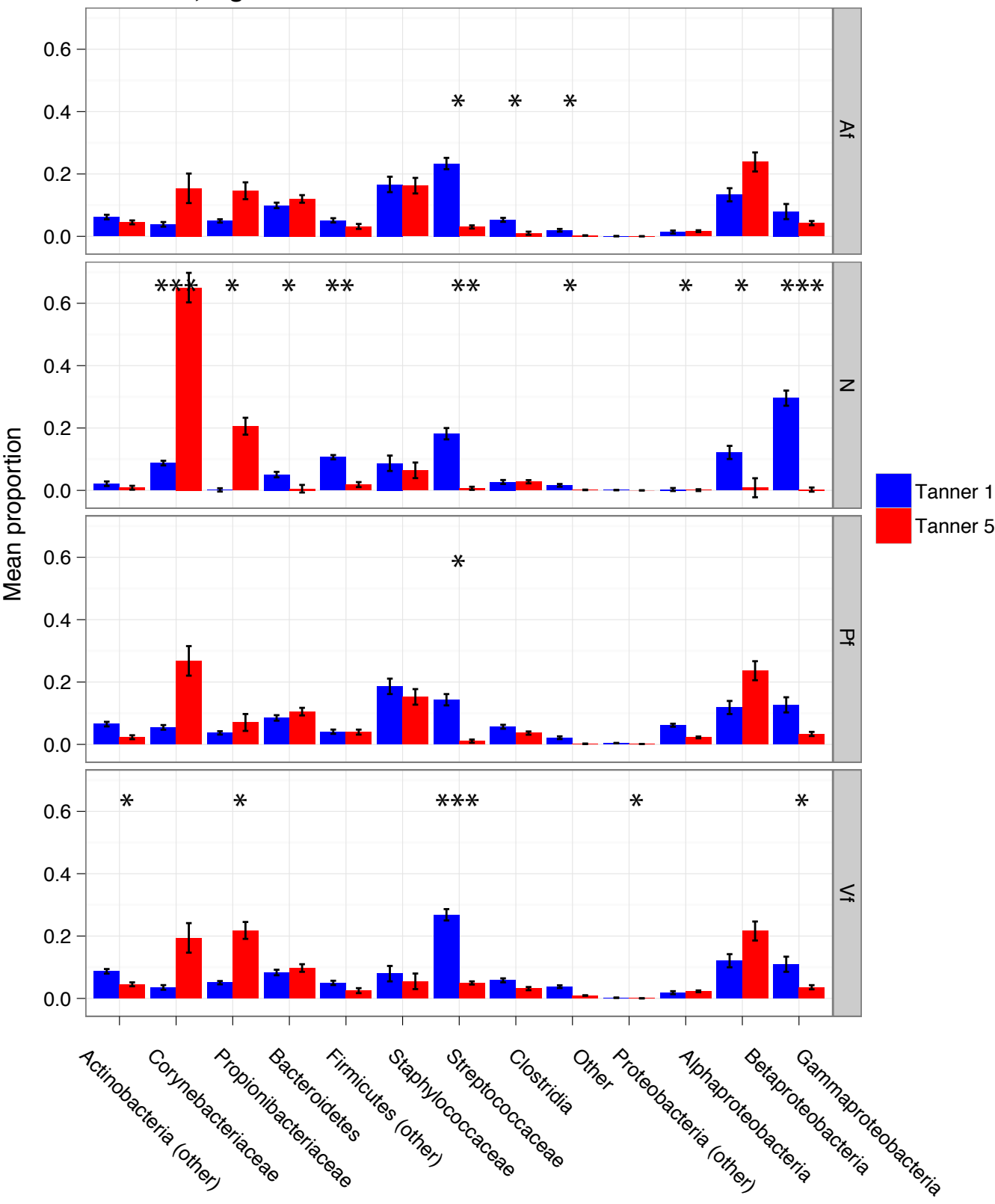

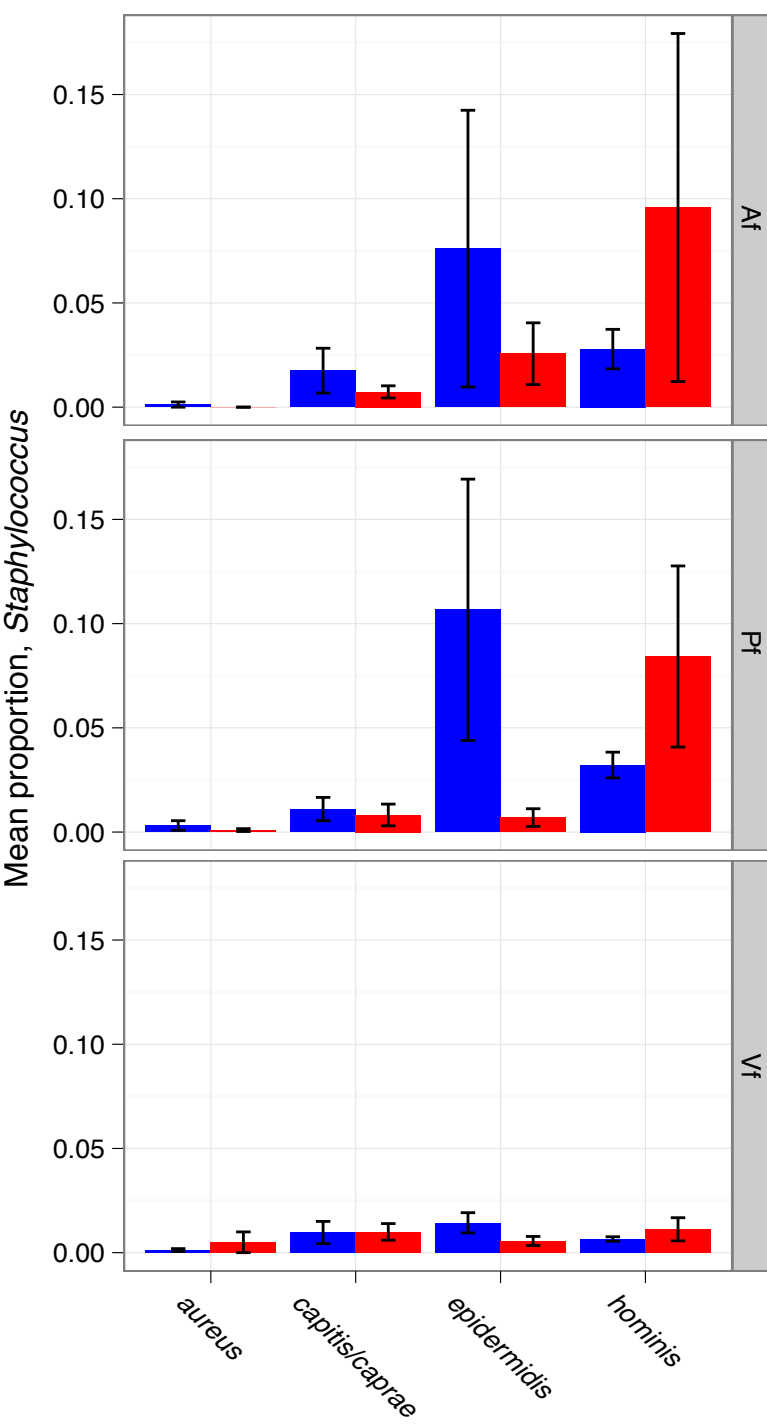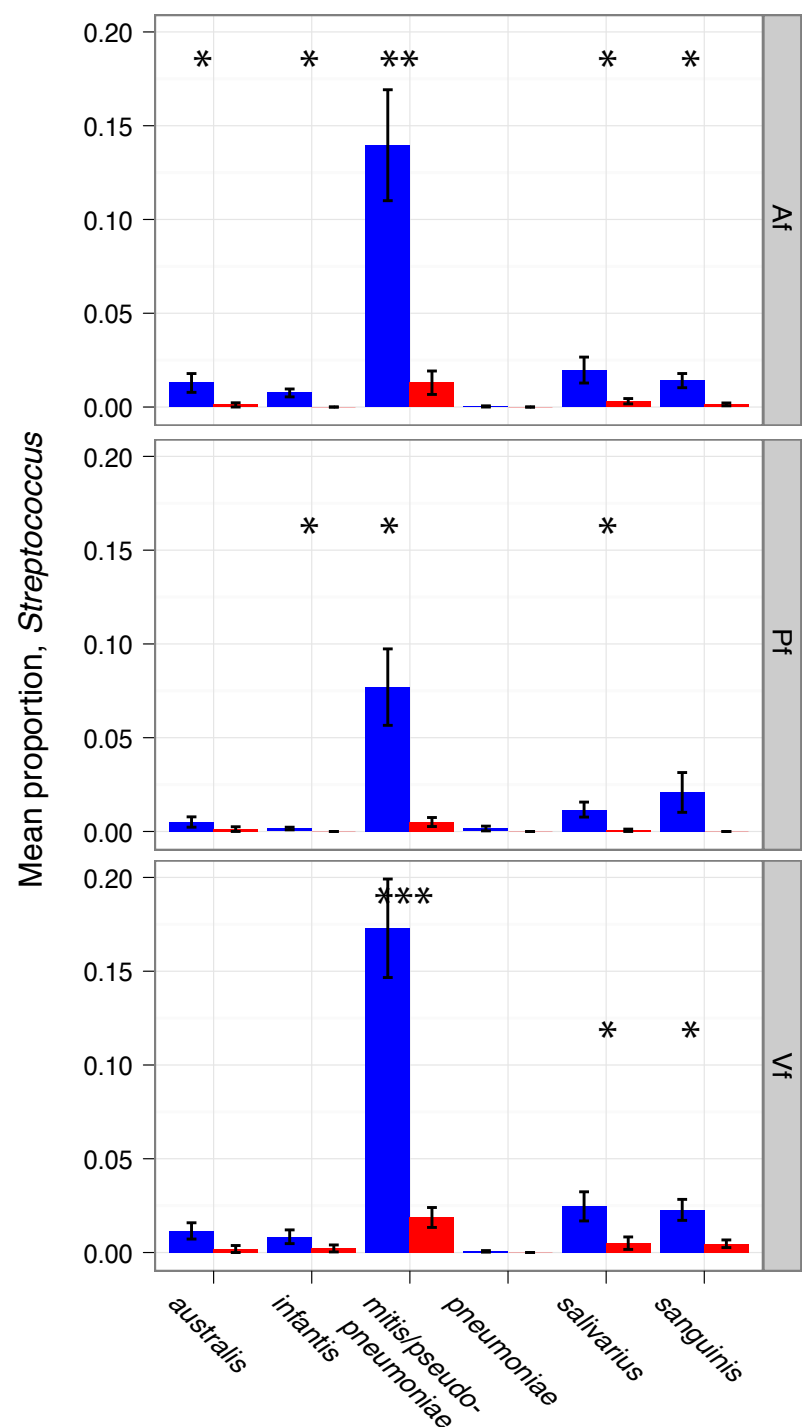

Supplement: Additional file 2 — Additional Figures S1 to S5. Figure S1: rarefaction analysis for the skin microbiota sampling at each site (Af, Pf, Vf, and N) calculated as operational taxonomic units (OTUs) at a cutoff of 97% similarity. Each point represents mean ± standard error of the mean of all individuals at the specified site and Tanner stage. Figure S2: communities clustered using principal coordinates analysis (PCoA) of the theta 'similarity' coefficients at all sites. Biplot arrows indicate the five most significant unique consensus taxonomies contributing to variation along axis 1. Spearman correlations with axes and associated P-values are shown in Table S3 in Additional file 1. Sites indicated are: Af, antecubital fossa; Pf, popliteal fossa; Vf, volar forearm. The nares plot is shown in Figure 1a. Analysis of molecular variance (AMOVA) testing differences in centroids is indicated for both Tanner 1 versus Tanner 5 and 'Tanner1-3' versus 'Tanner4-5'. Figure S3: bacterial taxonomic classifications for additional sites. Tanner stage is indicated. Figure S4: mean relative abundances by Tanner group for 12 major phyla-family taxonomic classifications. *P ≤ 0.05, **P ≤ 0.001, ***P ≤ 0.0001. Tanner 1 in red; Tanner 5 in blue. Figure S5: mean relative abundances by Tanner group for major species designations for genera Streptococcus and Staphylococcus. *P ≤ 0.05, **P ≤ 0.001, ***P ≤ 0.0001. Tanner 1 in red; Tanner 5 in blue. [file gm378-S2.PDF]
